# Peri-implant soft tissue health and denture base adaptation of CAD/CAM milled versus 3D-printed implant-assisted mandibular overdentures: one-year randomized clinical trial

**DOI:** 10.1038/s41598-026-40422-9

**Published:** 2026-03-13

**Authors:** Thuraya Maher Elmanci, Khloud Ezzat Mourad, Sally Elsayed Abdelsameaa, Marwa Ahmed Aboelez, Radwa Mohsen Kamal Emera

**Affiliations:** 1 Department of Prosthodontics, Faculty of Dentistry, Mansoura University, Mansoura, Egypt; 2 Department of Prosthodontics, Faculty of Dentistry, Mansoura National University, Gamasa City, Egypt; 3 Department of Oral and Maxillofacial Surgery, Mansoura University, Mansoura, Egypt; 4 Department of Preventive and Restorative Dentistry, Faculty of dentistry, Applied Science Private University, Amman, Jordan

**Keywords:** 3D-printed overdentures, Milled overdentures, Ball attachment, Denture adaptation, Peri-implant health, Diseases, Health care, Medical research

## Abstract

This study aimed to compare denture base adaptation and peri-implant soft tissue health between CAD/CAM milled and 3D-printed mandibular implant-assisted overdentures. Twenty-four completely edentulous patients were enrolled and randomly allocated to two groups. After two dropouts, twenty-two patients completed the trial (11 per group). All patients received two implants in the mandibular canine region with ball attachments. The intervention group received a CAD/CAM milled mandibular overdenture, while the control group received a 3D-printed mandibular overdenture; both groups received a conventional maxillary complete denture. Peri-implant soft tissue health was assessed using clinical parameters, including Gingival Index (GI), Plaque Index (PI), Bleeding Index (BI), and Probing depth (PD) at insertion (T0), six months (T6), and one year (T12) after denture insertion. The adaptation of the denture base for both groups was assessed by digital surface matching software (Geomagic). Regarding peri-implant soft tissue health, clinical parameters increased significantly with time for both groups, and there was no significant difference in all parameters between the two groups at all observational times. The whole surface adaptation between the two groups was significantly different, with Group I showing significantly higher adaptation than Group II. Within the limitations of this study, CAD/CAM milled implant-assisted mandibular overdentures demonstrated superior denture base adaptation compared to their 3D-printed counterparts. This finding underscores the precision achievable with subtractive manufacturing for definitive prostheses. Regarding the peri-implant tissues, both groups maintained comparable and stable levels of plaque control and gingival health throughout the observation period.

*Clinical Trial Registration Number*: No. NCT06166446.

## Background

To address the functional inefficiencies associated with traditional dentures, implant-assisted overdenture (IOD) is a well-recognized treatment option^1,2^. The McGill Consensus Statement outlines that the minimum standard of care for edentulous individuals, particularly in the mandible, is an overdenture supported by two implants as the initial treatment approach^1^. The most common types of unsplinted attachments used to retain an overdenture with two implants are locator and ball attachments^3^. Of these, the ball-and-socket attachment is frequently favoured for maintaining a mandibular overdenture due to its simplicity and cost-effectiveness^3^. However, the stress transfer and distribution of occlusal pressure in two implant-assisted prostheses differ from those in conventional complete dentures^4^.

Functional stresses cause the implant to act as a rotating fulcrum, concentrating significant forces in the housing area of the attachment. This can lead to bone loss in the ridge area, as the mucosa covering the ridges is more resilient than the rigid implant attachments^5,6^. Therefore, one of the critical factors influencing the clinical performance of an implant-assisted overdenture is the adaptation of the denture^7^.

There are various techniques for the construction of complete overdentures, with each method aiming to achieve an optimal balance between minimal deformation, proper adaptation, biocompatibility, and aesthetic appeal^8–10^. Since 1936, a range of processing techniques has been developed and successfully implemented in conventional denture manufacturing processes^9^. One notable issue is the shrinkage that occurs during the polymerization of poly-methyl methacrylate (PMMA)^11^. However, the field of implant overdenture fabrication has advanced significantly with the introduction of digital technologies^8,9,12^. These technologies, driven by computer-aided design and manufacturing (CAD/CAM), include three-dimensional (3D) printing (additive manufacturing) and milling (subtractive manufacturing)^8,12–14^. With the continued advancement of digital technologies, it is anticipated that the complicated procedures associated with traditional denture manufacturing techniques will be overcome^8,10,12^.

One additive manufacturing technique, vat photopolymerization, involves solidifying a liquid resin layer by layer using an ultraviolet light source through a process called photopolymerization^15,16^. In contrast, subtractive manufacturing involves milling a prefabricated block to achieve the desired shape, using digital images as a guide^16,17^. The fabrication of dentures using computer-aided design and computer-aided manufacturing (CAD-CAM) is particularly noteworthy because it simplifies the process and reduces the number of laboratory steps required^16,18^. Digital design also ensures that the denture base maintains a consistent thickness, which can be adjusted to a minimum to ensure patient comfort. However, it is essential to note that techniques like 3D printing are susceptible to inherent artifacts, such as post-polymerization shrinkage and surface roughness resulting from the stair-stepping effect, which can affect the final fit. Consequently, process control to minimize deformation, from design to polymerization, is critical to realizing the potential for superior denture adaptation^16,19^.

The health of peri-implant soft tissues is a critical determinant of long-term implant success^20,21^. For implant-supported overdentures, both the choice of prosthesis materials and the construction technique can significantly influence the peri-implant environment, affecting plaque accumulation, soft tissue inflammation, and the risk of peri-implant diseases. The literature, however, presents varying findings and ongoing debate regarding the impact of different materials and fabrication methods on soft tissue response.

Well-adapted dentures offer several advantages, including improved chewing efficiency, acceptable retention, stability, and comfort^7^. Using superimposition analysis of STL files from scanned denture bases, several studies have investigated the adaptation and clinical performance of mandibular CAD-CAM complete dentures in comparison to conventional complete denture bases^22–25^. Moreove While traditional over, most of these studies are in vitro^22,25^. Clinical research on denture base adaptation and the peri-implant soft tissue health of CAD/CAM milled versus 3D printed implant-assisted mandibular overdentures is limited. Therefore, this study aimed to compare the denture base adaptability and the peri-implant soft tissue health between CAD/CAM milled and 3D printed implant-assisted mandibular overdentures.

The primary null hypothesis of this clinical trial postulated that there would be no significant differences in the soft tissue health surrounding implants between CAD/CAM milled implant-assisted overdentures and those fabricated through 3D printing techniques. Furthermore, the secondary null hypothesis proposed that there would be no differences in the adaptation of denture bases resulting from the two manufacturing methods.

## Methods

### Participant selection and study design

All study methods were conducted in accordance with relevant guidelines and regulations, as the study protocol received ethical approval from the Ethics Board Committee of the Faculty of Dentistry (No. A10080921, and written informed consent was obtained from all participants prior to enrollment. Additionally, the study protocol was registered at clinicaltrials.gov (No. NCT06166446).

The present randomized clinical trial study was conducted on completely edentulous patients who were unsatisfied with their mandibular conventional dentures and were referred to the prosthodontic department, Faculty of Dentistry, for oral rehabilitation by implant-assisted mandibular overdenture. The sample size for this study was calculated on the G-power program (version 3.1.9.7). Depending on the value of the effect size (f), which = 1.32, obtained from a previous study^24^. Assumed that the maximum level of acceptable error is 5% (i.e., d = 0.05), and the power of estimation = 80%, and there were 2 groups of participants in a comparison to test significant differences in denture base adaptation (labial flange) through an independent t-test. (Conventional = 0.011 ± 0.015) and (3D printed = 0.078 ± 0.070). The minimum required sample size was calculated to be 22 participants (11 per group). To account for a potential 10% dropout rate, 24 participants were recruited and enrolled in the study. Twenty-two participants (14 males and 8 females, aged 45–80 years, mean age 63 years) ultimately completed the trial and were included in the final analysis.

Participants were selected based on several criteria: the maxillary and mandibular alveolar ridges exhibited firm, healthy, evenly compressible mucosa free from inflammation; patients were completely edentulous with a minimum alveolar ridge width of 6 mm and a minimum bone height of 13 mm in the inter-foraminal area, verified by 3D panoramic analysis, to ensure the safe placement of the smallest implant used in the study (3.5 mm diameter x 11.5 mm length); there was adequate inter-arch space as indicated by preoperative tentative jaw relation; patients expressed dissatisfaction with their mandibular dentures due to stability and retention issues; and all patients had maxillo-mandibular relationships classified as Angle’s Class I. Exclusion criteria included systemic conditions contraindicating implant placement, TMJ disorders, neuromuscular diseases, bone metabolic disorders such as diabetes, smoking, and parafunctional habits.

### Randomization, allocation concealment, and blinding

The participants were randomly allocated to one of the two groups (1:1 ratio) using a computer-generated random number sequence (Microsoft Excel). The allocation sequence was concealed from the researcher (T.M.) who enrolled and assessed participants. The assignment was implemented by a dental practitioner not involved in the study, who opened sequentially numbered, opaque, sealed envelopes containing the group assignment. The examiner responsible for all clinical peri-implant measurements was blinded to the group assignment throughout the study. Due to the nature of the interventions, the participants and the prosthodontists providing care could not be blinded to the group assignment. However, the clinician performing the peri-implant soft tissue assessments (S.A.) and the operator analyzing the denture base adaptation (T.M.) were blinded to the group allocation.

**Fig. 1:**
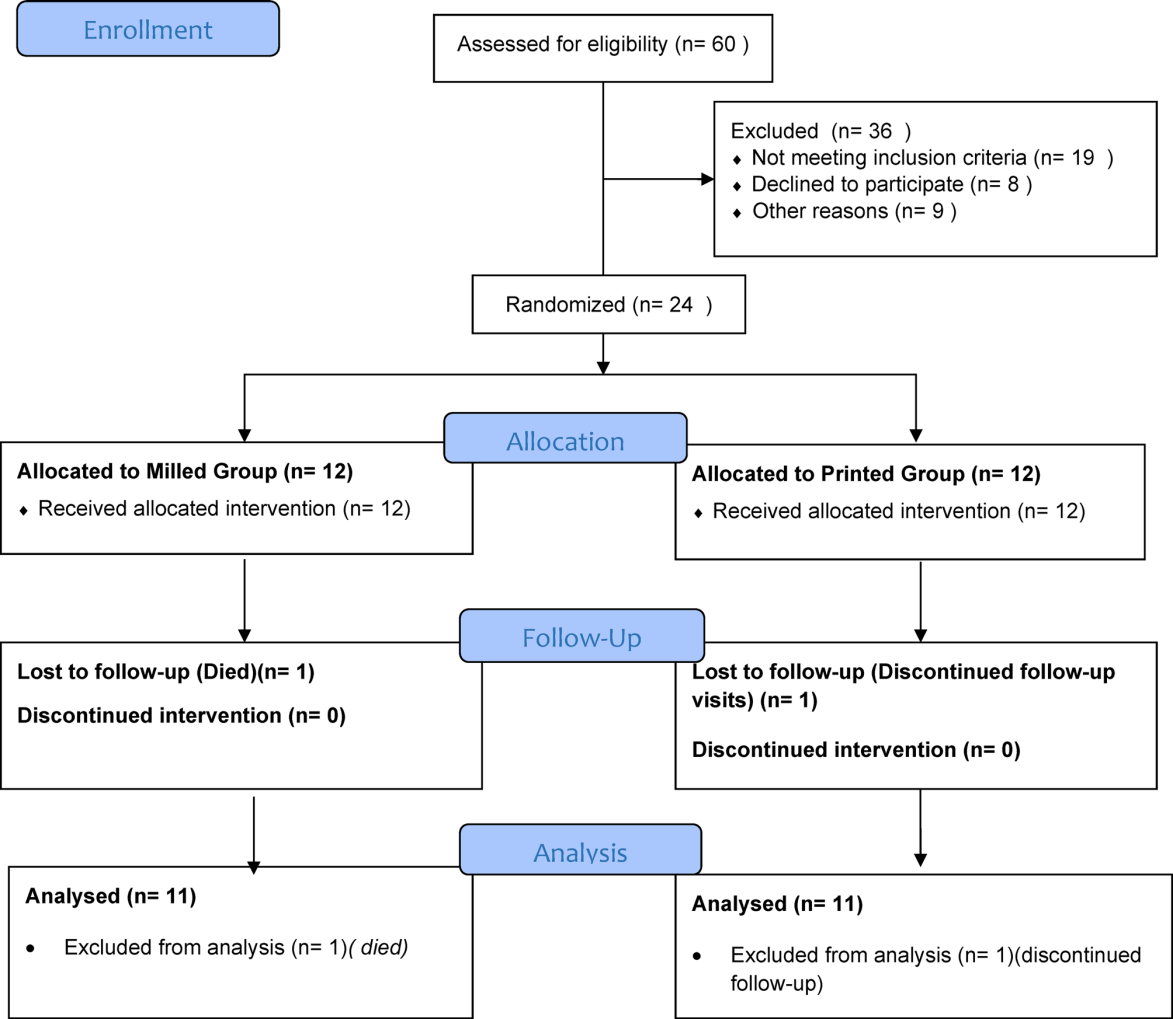
CONSORT 2010 flow diagram.

The two groups are: Group I (Milled): Participants received a maxillary conventional acrylic complete denture and a CAD/CAM milled implant-assisted mandibular overdenture (*n* = 12). Group II (3D-Printed): Participants received a maxillary conventional acrylic complete denture and a 3D-printed implant-assisted mandibular overdenture (*n* = 12) (Fig. 1).

### Surgical and prosthetic procedures for both groups

New maxillary and mandibular conventional dentures were constructed for each patient. Additional steps were taken during the construction process to prepare the STL files needed for the fabrication of milled and 3D-printed dentures. These steps included scanning the trial denture bases mounted on the articulator to register the vertical dimension and centric relation, as well as scanning the polished surfaces and occlusal relationships of the final denture using a 3D scanner (Medit i500, Korea). This process ensured the duplication of the shape, form, and size of the teeth from the conventional denture in the newly constructed digitally designed mandibular overdenture.

The mandibular denture was duplicated in heat-cured acrylic resin to create a radiographic template. Gutta-percha markers were placed on the lingual and buccal surfaces of the duplicated dentures. Using Cone Beam Computed Tomography (Vatech, Seoul, Korea) and a dual scan technique, a three-dimensional image of the edentulous mandible was constructed with digital planning software (OnDemand3D, Cybermed Inc., Seoul, Korea). Two straight implants (Neobiotech dental implant, IS-II active fixture) were virtually planned to be parallel in the canine regions. A mucosal-supported stereolithographic surgical guide was created, featuring guided openings for the implants and anchor pin installation, using rapid prototyping machinery (In2Guide).

Patients rinsed with chlorhexidine digluconate mouthwash two weeks before surgery. A peri-operative antibiotic regimen of Augmentin (1 g, comprising 875 mg amoxicillin and 125 mg clavulanic acid) was administered, starting the day before surgery and continuing twice daily for seven days postoperatively. This protocol was intentionally selected to mitigate the risk of early implant failure and peri-implantitis during the critical early healing phase. Given that the study’s primary outcome was peri-implant soft tissue changes, this conservative approach was deemed necessary to minimize the potential confounding variable of post-operative infection in this specific patient cohort with a history of long-term edentulism and denture wear.

**Fig. 2:**
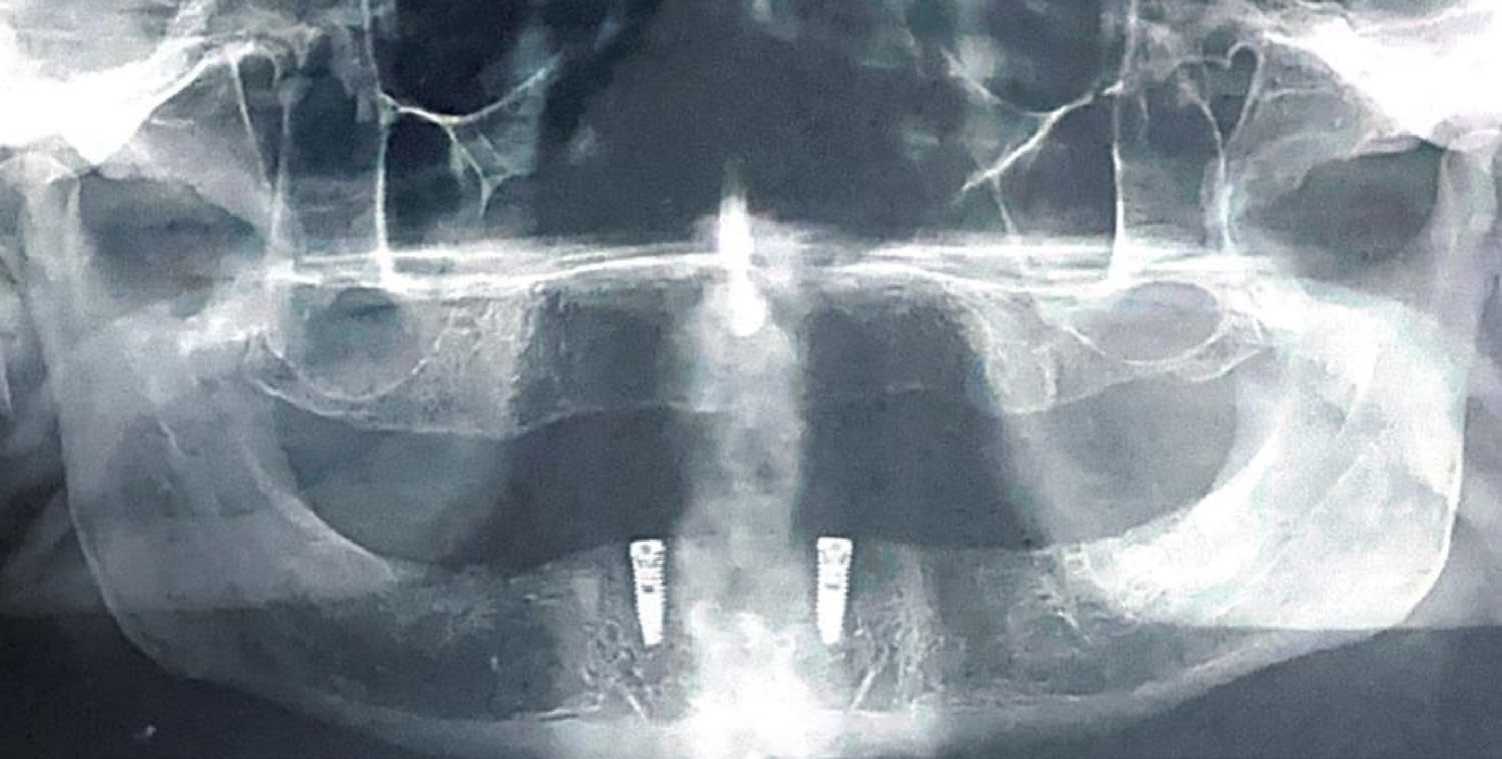
Panoramic x-ray for verification of orientation and position of implants.

Using a flapless technique and delayed loading protocol, two implants (11.5 mm length, 3.5 mm diameter) were placed. A surgical stent was secured to the bone with anchor pins to guide the implants, which were inserted with a computer-guided surgical kit (On Demand, South Korea). Post-surgery, panoramic radiographs assessed implant positioning (Fig. 2). The patient’s complete denture was relined using soft liner (Promedica, Germany) and delivered as an interim restoration right after the implants were placed.

After a three-month osseo-integration period, healing abutments (4 mm diameter and 3 mm height) were screwed onto each fixture for two weeks to facilitate the healing of the peri-implant tissues. An implant-level impression was made for both groups using the open-tray impression technique, as described by E. Jorge et al.^26^Following the removal of the healing abutments, long transfer copings were attached to the implants. Light-body rubber base material was injected around the transfer copings, and a loaded tray containing heavy-body impression material was positioned. Once the impression material had set, the long transfer copings were unscrewed, and the impression was carefully removed. Implant analogues were then screwed into the transfer copings.

After pouring the master cast, 3.5 mm diameter and 2 mm height ball abutments (Neobiotech) were attached to the implant analogues, with their metal housings securely fitted. The master cast was scanned extra-orally using a 3D scanner (Medit i500, Korea) to create an STL file. This file was imported into the design software as the foundation for the new digital overdenture. To ensure the new prosthesis replicated the successful aesthetics and function of the existing one, key parameters were extracted from the previously obtained scan of the conventional denture and applied to the new design. Specifically, the tooth position, arch form, polished surface contours, and the pre-registered vertical dimension of occlusion from the conventional denture were used as a digital reference guide. The technician utilized these parameters while digitally sculpting the new denture base onto the scanned master cast. The virtual design for the mandibular implant-supported overdenture base, which included integrated cavities for the attachment housing and their escape vents on the fitting surface, was then finalized and approved. A try-in step was subsequently performed using a resin prototype of the mandibular digital overdenture.

**Fig. 3:**
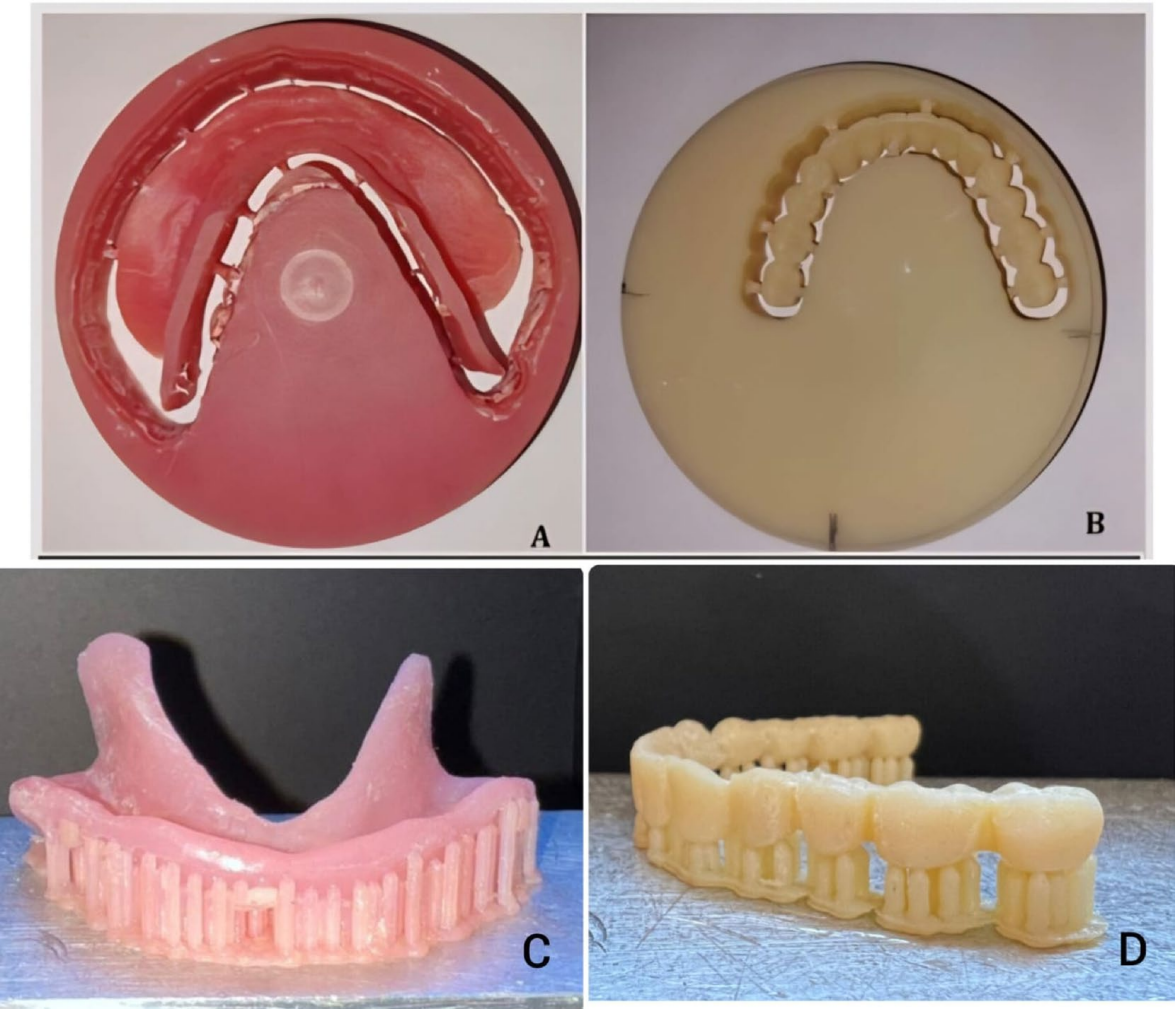
(**A**) Milled denture base before removing supports. (**B**) Milled denture teeth before removing supports. (**C**) 3-D printed denture base with supporting frame.

The digital design of both milled and 3D-printed overdentures was performed using the manufacturer’s recommended CAD/CAM workflows. No user-defined offset or compensation values were manually applied. For the milled dentures, compensation parameters were automatically managed by the milling software according to Ivoclar’s validated protocol. For the 3D-printed dentures, shrinkage compensation was automatically applied by the printer software based on the resin manufacturer’s default settings. The final prosthesis construction was completed according to the patient group assignments after verification of the resin try-in (Fig. 3). For Group I, patients received milled mandibular overdentures. The denture base was milled from a pre-polymerized polymethylmethacrylate block (Ivobase CAD, Ivoclar Vivadent, Schaan, Liechtenstein) and the denture teeth were milled from a tooth-colored PMMA block (SR Vivodent CAD, Ivoclar Vivadent). For Group II, patients received 3D-printed mandibular overdentures. 3D Printing Protocol: The definitive overdentures for Group II were fabricated using Digital Light Processing (DLP) technology with a Rasedent D50 3D printer (Rase Dent Co., Ltd., Korea; build volume: 119 × 67 × 75 mm). The denture bases were printed using a dedicated photopolymer resin for denture bases (DENTCA Denture Base II, DENTCA, Inc., Torrance, CA, USA) at a layer thickness of 50 μm, using a 405 nm LED light source. The build orientation was set to 0° (platform parallel). Supports were generated automatically using the printer software’s default settings for denture bases. After printing, supports were removed, and the bases were washed in 99% isopropanol for 3 min and post-cured in a UV chamber (CureM, Rase Dent) at 60 °C for 15 min. The mandibular denture teeth were printed as individual units from a tooth-shaded resin (Dentca Denture Teeth resin, shade A2, Dentca Inc., Torrance, CA, USA) and subsequently bonded to the cured denture base using a dedicated light-polymerizing denture repair resin (DENTCA Denture Base II, DENTCA, Inc., Torrance, CA, USA). The bonding surfaces were first cleaned with isopropyl alcohol and lightly abraded with a micro-etcher. A thin layer of resin was applied, the tooth was positioned under slight pressure with reference to the approved try-in, and the resin was light-cured for 20 s per surface according to the manufacturer’s instructions.

**Fig. 4:**
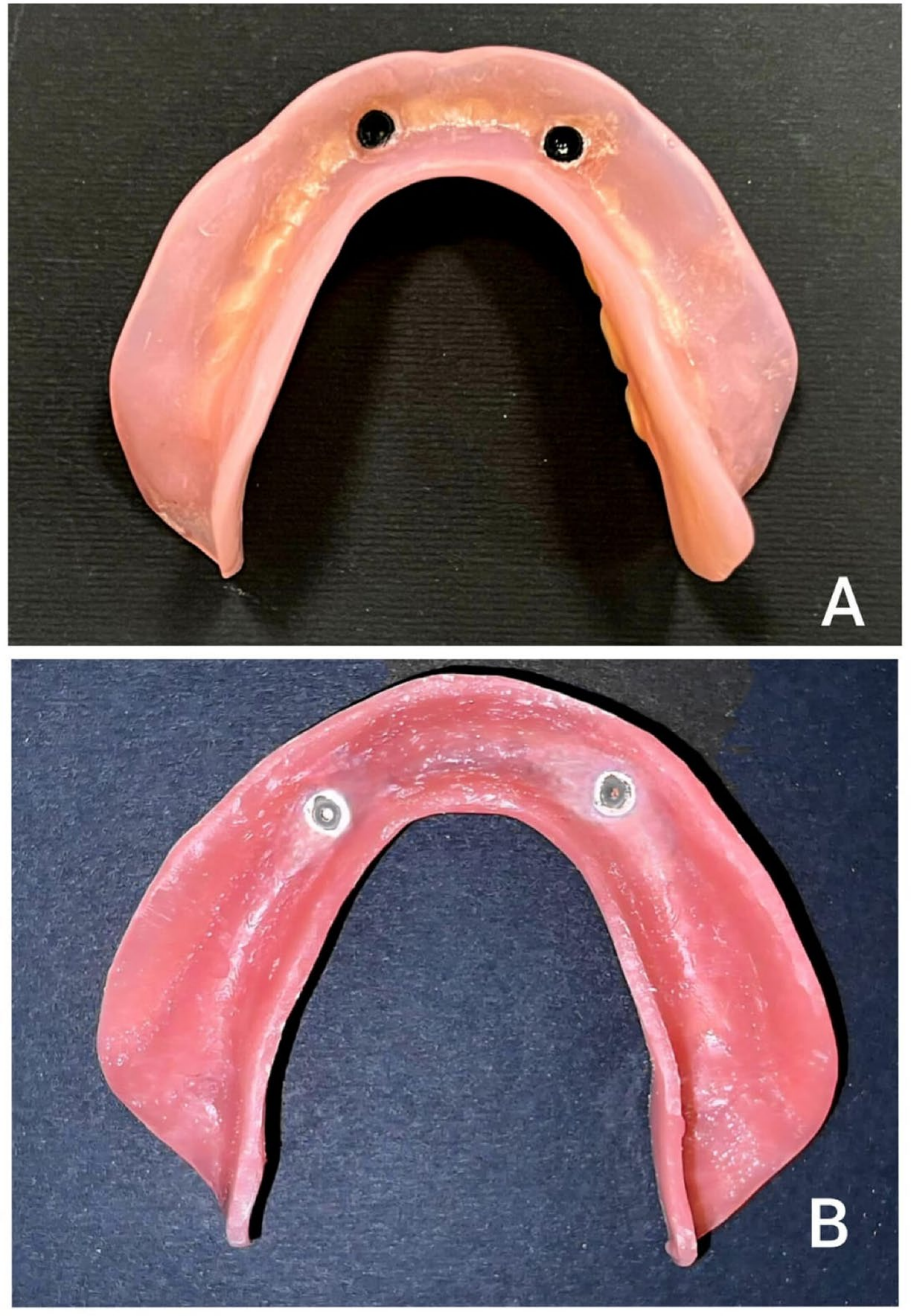
Close-up view of the fitting surface illustrating the extraoral pick-up of the metal attachment housing within the denture base using autopolymerizing resin. A: 3D-printed mandibular overdenture. B: Milled mandibular overdenture.

**Fig. 5:**
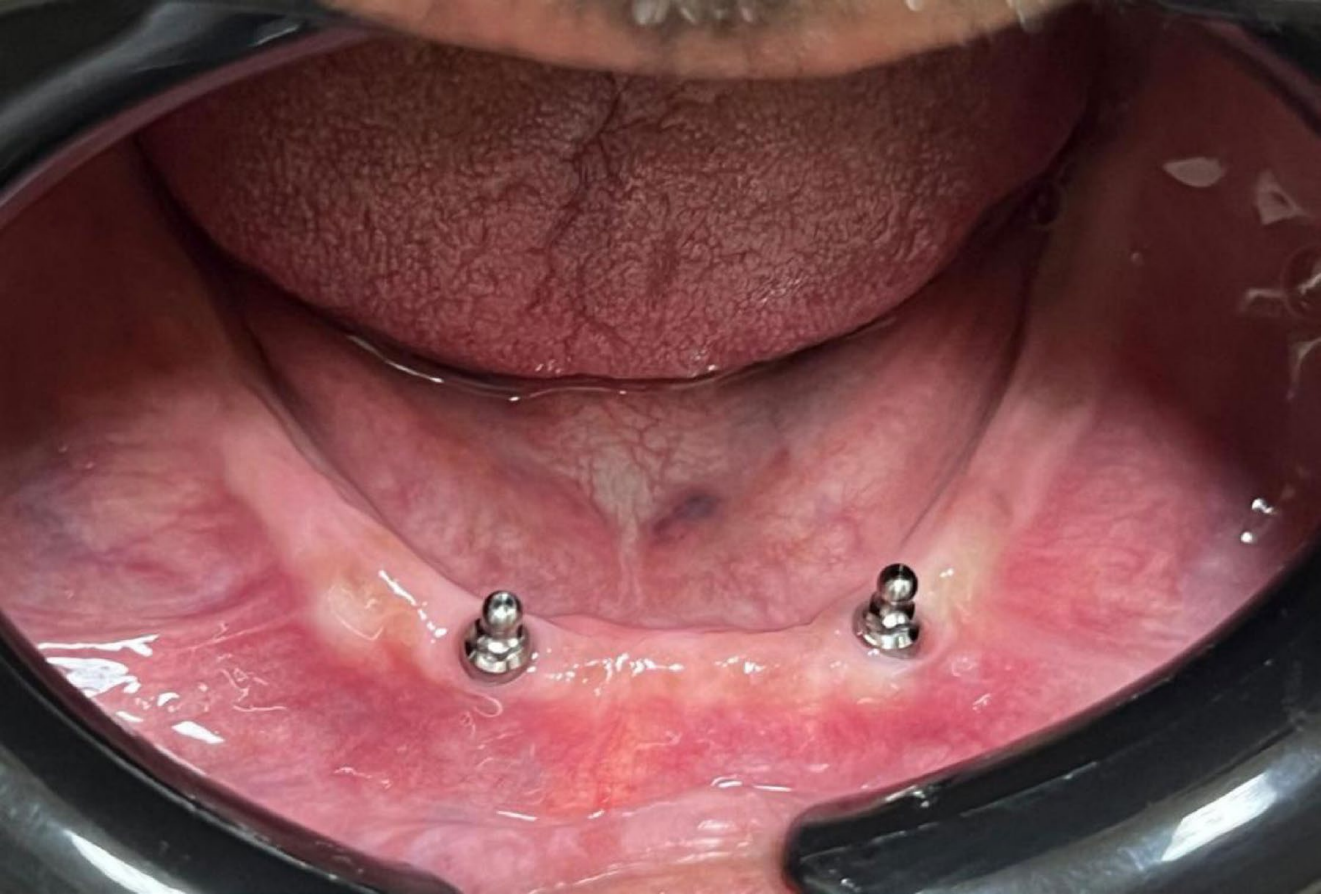
Ball attachments screwed to implants.

**Fig. 6:**
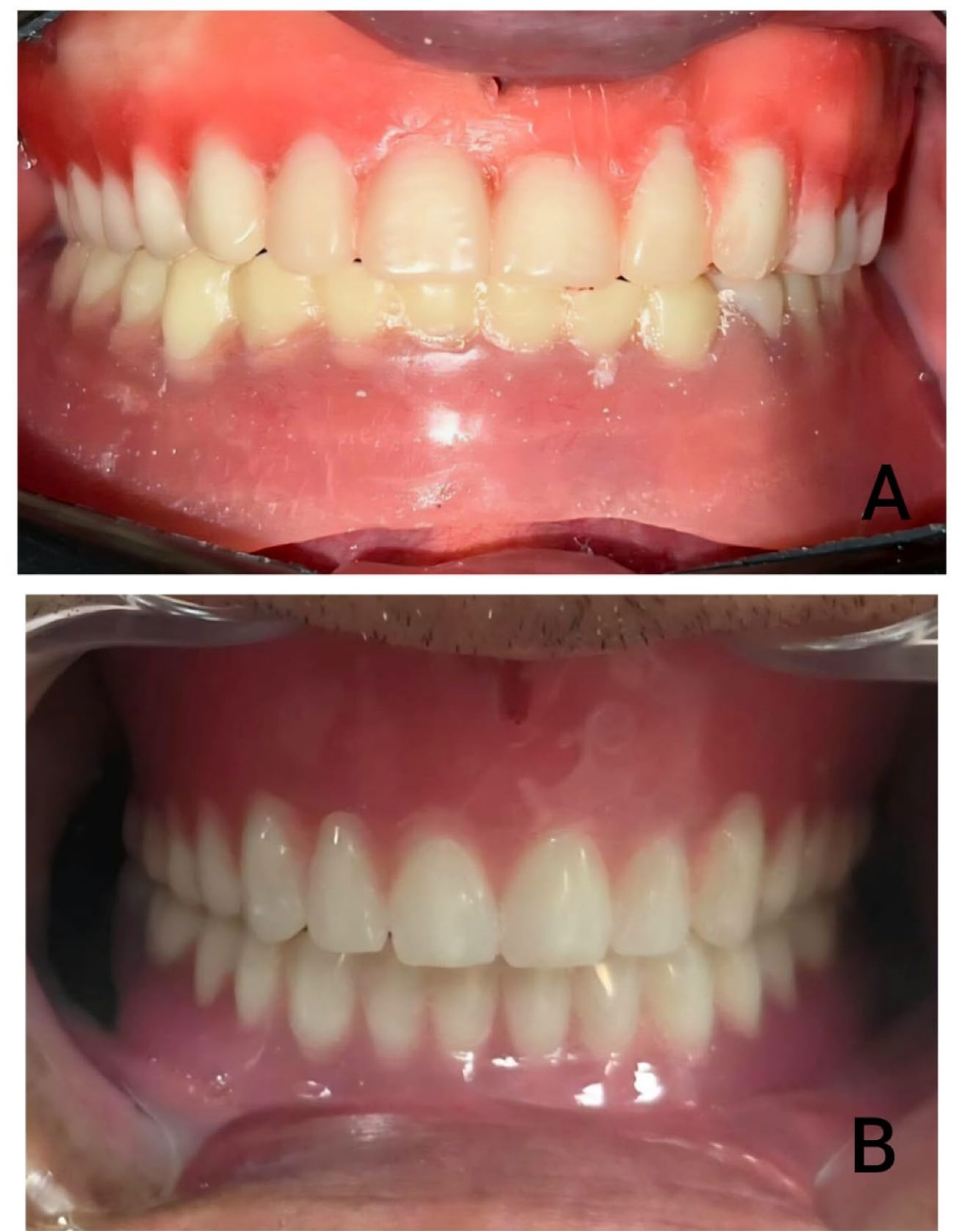
Clinical and technical photographs of the implant-assisted overdentures. (**A**) Intraoral view of the CAD/CAM milled mandibular overdenture in occlusion with the conventional maxillary denture. (**B**) Intraoral view of the 3D-printed mandibular overdenture in occlusion.

For both groups, the metal housings for the ball attachments were picked up extra-orally into their designated cavities on the fitting surface of the denture base using auto-polymerizing resin (Duralay, Reliance Dental Mfg. Co., Worth, IL, USA) (Fig. 4) This extraoral method was chosen to avoid the potential complications of intraoral pickup, such as resin flow into the peri-implant sulcus and patient discomfort during polymerization^27,28^. This was performed on the master cast, which accurately replicated the intraoral implant positions. The master cast was mounted on a semi-adjustable articulator (Bio-art A7plus, Jd. Tangara-SP) using the pre-registered jaw relation records, ensuring the preservation of the established vertical dimension of occlusion and occlusal relationships. Excess material was removed, and the denture base was finished and polished. The plastic retentive inserts were fitted into their housings, and the dentures with attachments were inserted after screwing the ball attachment intraorally (Fig. 5). The occlusion for all definitive overdentures was adjusted intraorally according to the lingualized occlusion concept^29^. This scheme was selected as it is widely recommended for complete dentures and implant-supported overdentures because it combines the stability of a bilaterally balanced occlusion with the simplicity and food-penetration advantages^30,31^. It directs occlusal forces more vertically along the long axis of the implants, which is considered beneficial for peri-implant biomechanics^32,33^. Both the esthetics and phonetics of the prostheses were evaluated and approved by the patients (Fig. 6).

## Peri-implant soft tissue evaluation

Follow-up appointments were scheduled at three months (T0/baseline after prosthesis insertion), 6 months (T6), and one year (T12) to evaluate peri-implant soft tissues. All assessments were performed by a single, calibrated examiner who was blinded to the patient’s group assignment. The following indices were recorded: Modified Plaque Index (PI) and Simplified Gingival Index (GI), as recommended by Mombelli et al.^34^. Modified Bleeding Index (BI): recorded as present (1) or absent (0) within 15 s after gentle probing^34^. Probing Depth (PD): measured in millimeters using a calibrated, pressure-sensitive plastic periodontal probe (Vivacare TPS, Ivoclar Vivadent, Schaan, Liechtenstein). The distance from the marginal border of the peri-implant mucosa to the tip of the probe was recorded with a light, standardized force^34^. Measurements for PI, GI, BI, and PD were assessed at the mid-buccal, mid-lingual, and the mesial and distal aspects of each implant. The mean PD per implant was calculated for statistical analysis.

### Assessment of denture base adaptation

**Fig. 7:**
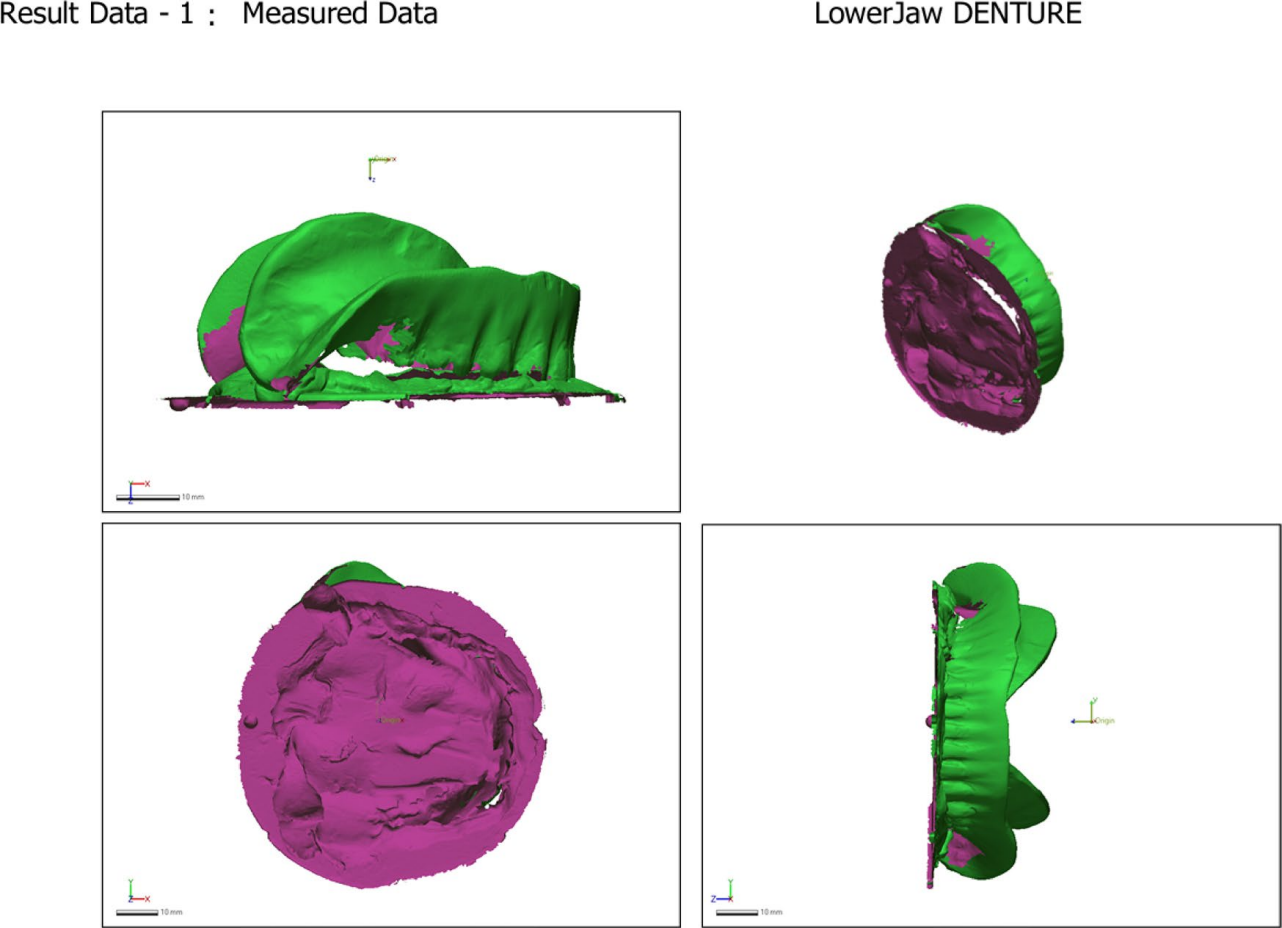
Scanned datasets: the STL files of the definitive master cast and the denture base fitting surface before alignment.

**Fig. 8:**
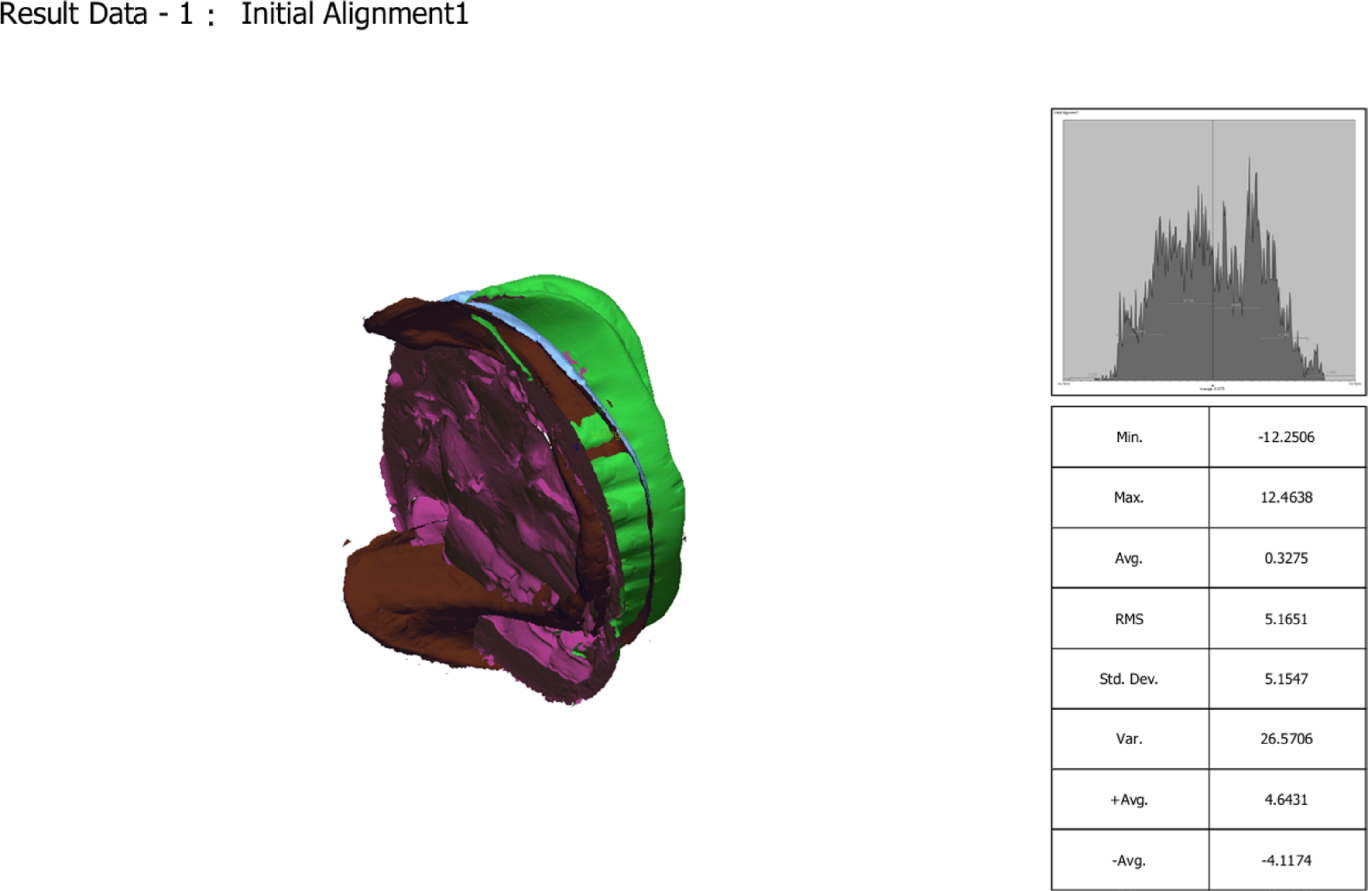
Initial alignment: the two models are coarsely aligned within the software.

**Fig. 9:**
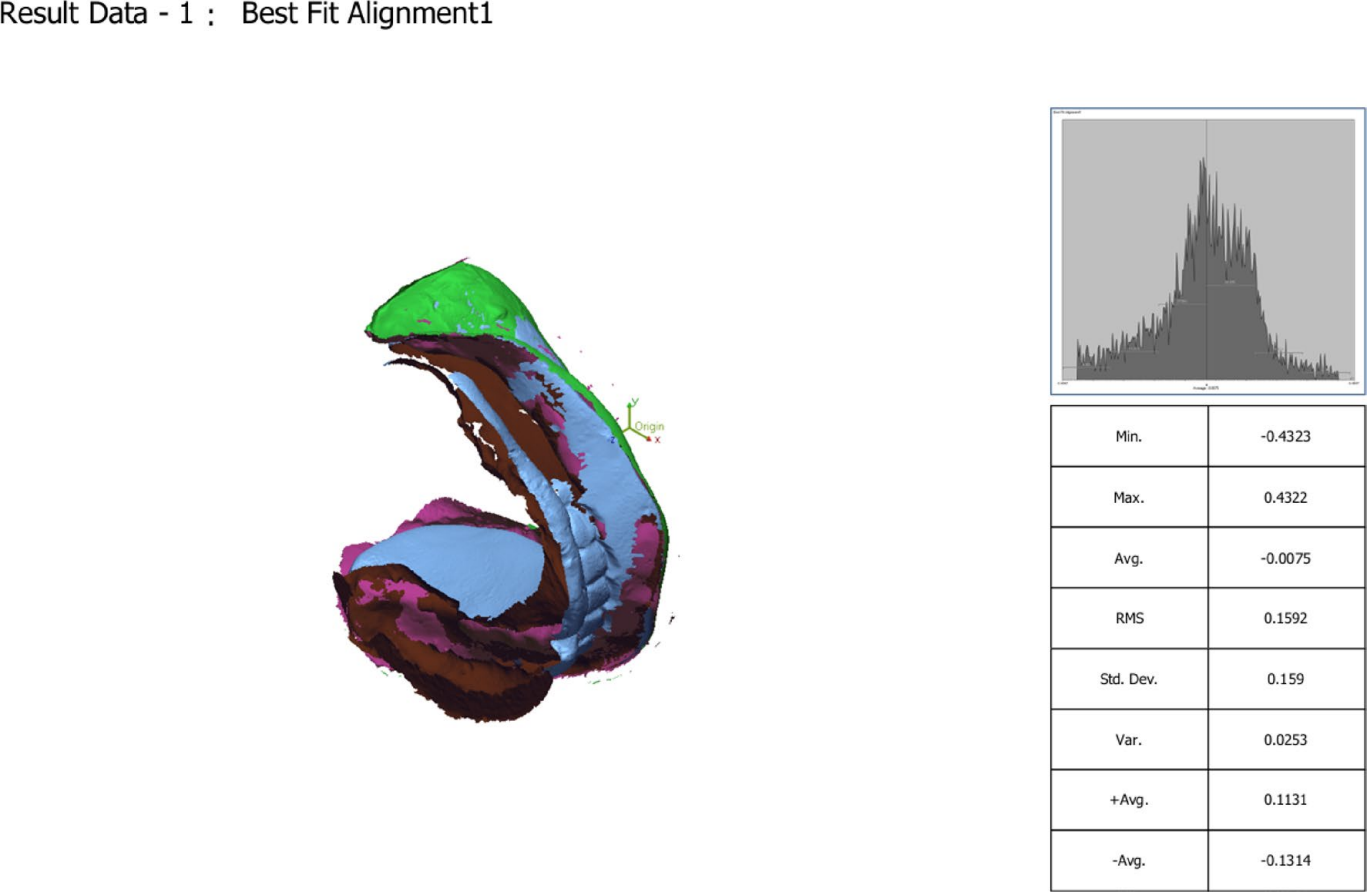
Best-fit alignment: final superimposition using the iterative closest-point algorithm in Geomagic Control X, restricted to stable anatomical regions (lingual vestibule and retromolar pads) for stable registration.

The adaptation of the definitive mandibular overdenture bases was assessed digitally after their fabrication but before clinical insertion. For this, the definitive master cast (with implant analogues) was scanned to create a reference STL file. Separately, prior to extraoral scanning with the 3Shape desktop scanner, the fitting surfaces of the lower denture bases were lightly coated with an anti-reflective titanium dioxide scanning spray (Digiscan-Spray, Art.-Nr. 581 − 0300, Yeni Dental Digital 1000, Istanbul, Turkey). The powder was applied from a distance of approximately 20–30 cm using short, even bursts to achieve a thin, uniform matte layer, avoiding pooling or excess. This standardized protocol was followed for all scans to ensure consistent optical properties and measurement reliability. To flip the fitting surface of the denture base, an STL file was exported and imported into Meshmixer. (Fig. 7) The initial alignment was checked (Fig. 8). Then, using the best-fit alignment option in the surface matching software (Geomagic Control × 64), the STL file of the denture base fitting surface was superimposed onto the STL file of the corresponding definitive cast (Fig. 9). A 3D deviation analysis was performed to generate a color-coded heat map. A tolerance threshold of ± 0.1 mm (100 μm) was selected. This value is supported by two factors: it represents twice the 50 μm layer thickness of the printing process, and it falls within the 50–100 μm tolerance band commonly reported in comparable dental metrology studies for assessing the accuracy of prostheses and implant components, where such deviations are often considered within clinically acceptable limits^35–39^.

### Digital superimposition and analysis protocol

**Fig. 10:**
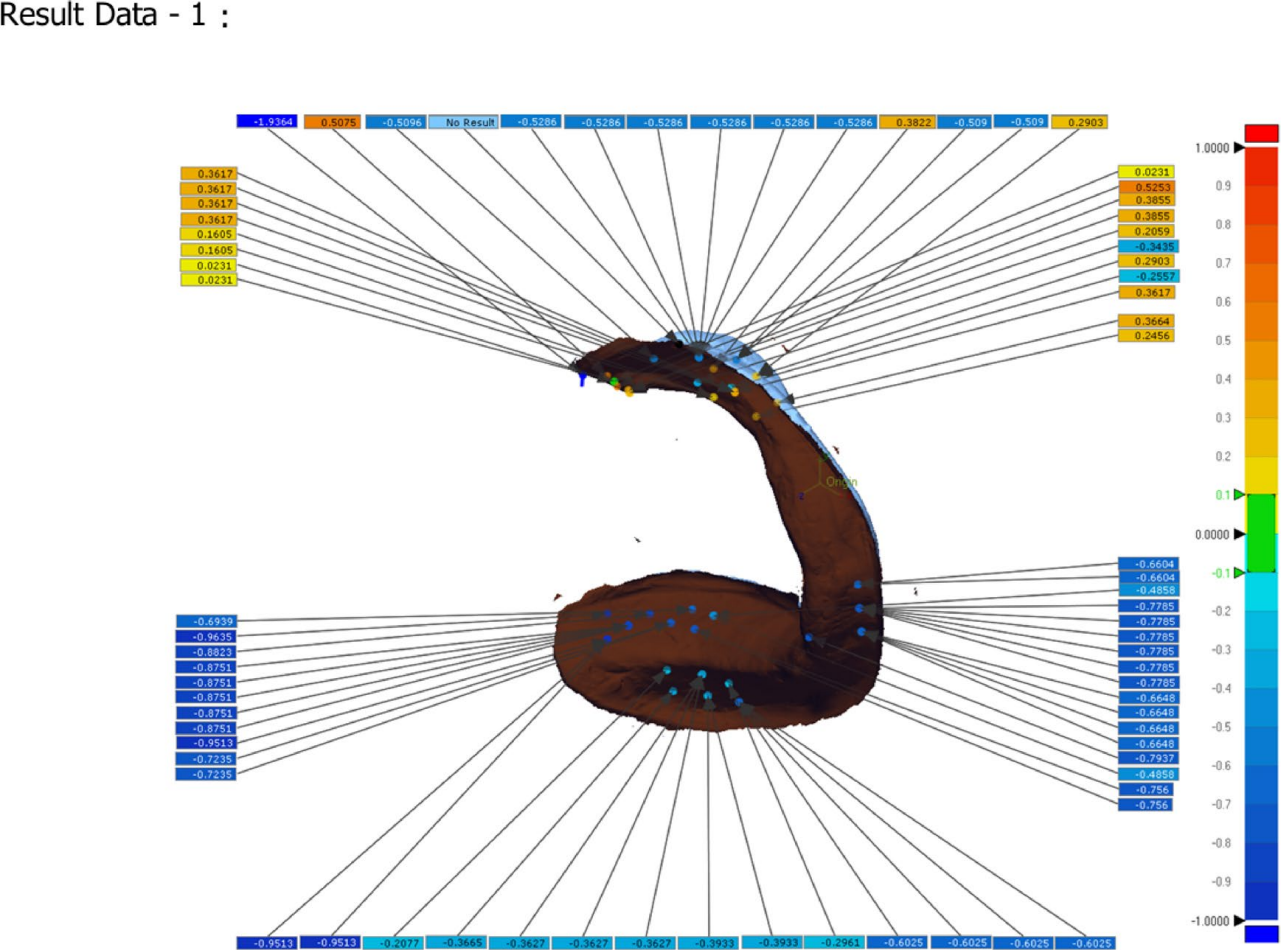
3D deviation analysis (heat map): Resultant color-coded map showing the signed distance between the surfaces. blue indicates negative deviation (denture inside the cast = interference/pressure), Yellow/Red hues indicate positive deviation (denture outside the cast = gap/space), and green indicates values within the ± 0.1 mm tolerance zone.

The STL file of the denture base fitting surface was aligned (superimposed) onto the STL file of the corresponding definitive master cast using the best-fit alignment algorithm in Geomagic Control × software (Fig. 10). This algorithm performs a global, iterative closest-point alignment, minimizing the root mean square error between the two entire surfaces. To ensure a stable and accurate alignment, the region of interest for the initial best-fit was restricted to stable anatomical areas away from the implant sites, specifically the mandibular lingual vestibule and the retromolar pad regions. This method is standard for aligning objects lacking discrete fiducial markers.

**Fig. 11:**
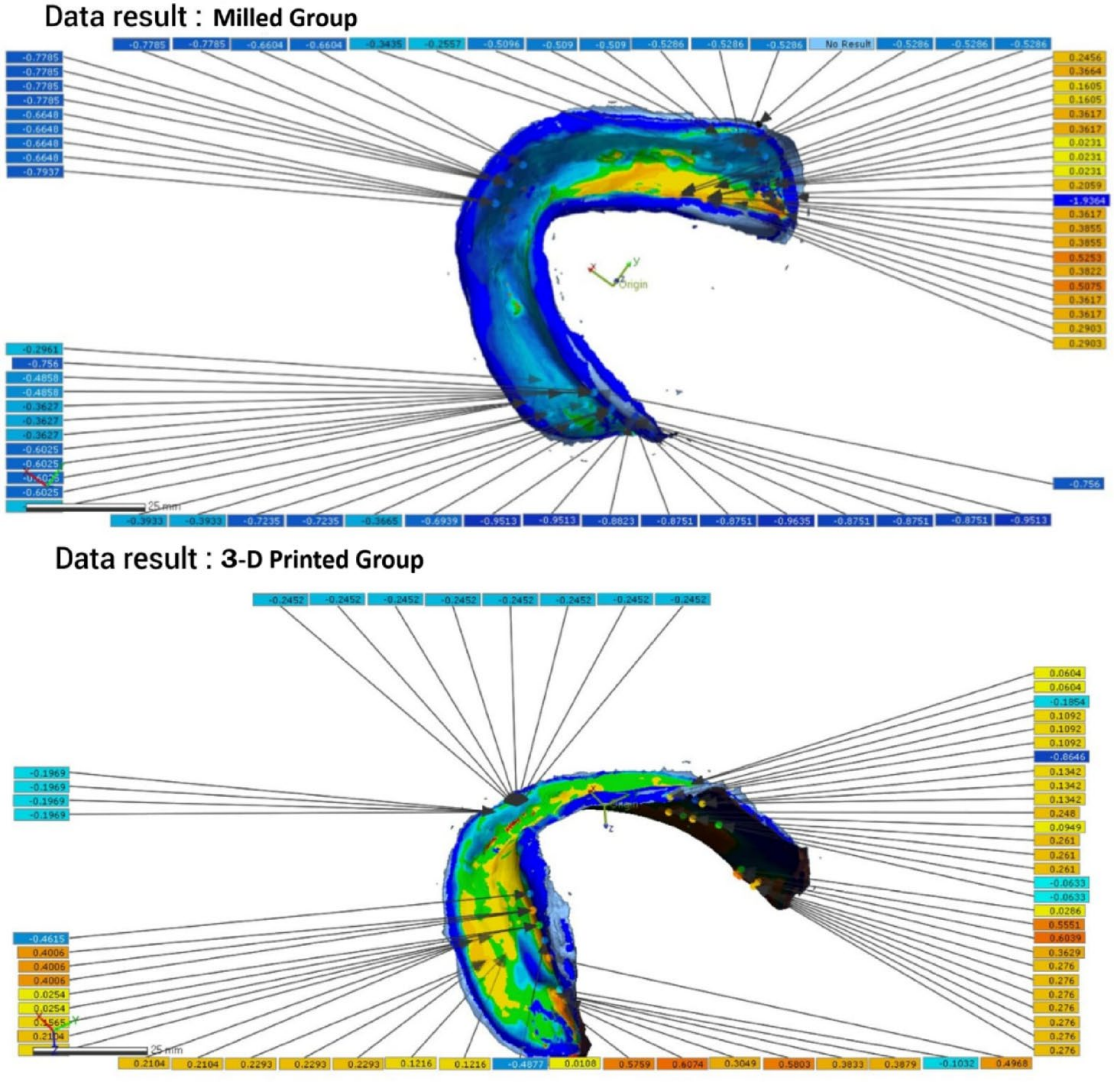
Using Geomagic Control × software, a three-dimensional color deviation map of a mandibular overdenture base superimposed on its definitive master cast. The color scale represents the signed distance (in mm) between the two surfaces. Blue hues (light to dark) indicate negative deviation, where the denture base is positioned inside the cast surface, representing interference or pressure. Yellow to red hues indicate positive deviation, where the denture base is outside the cast surface, representing a gap or space. Green indicates minimal deviation within the defined clinical tolerance zone (± 0.1 mm).

Following this superimposition, the 3D Compare function was used to perform a full-surface deviation analysis. For a detailed regional assessment, values were recorded at nine pre-defined points: the right and left buccal shelf (RBF, LBF), right and left lingual flanges (RLF, LLF), right and left retromolar pads (RRP, LRP), and the labial flange (LF). First, the 3D Compare option was utilized to examine the entire lower cast surface, assessing how well the measured data adapted to it. This assessment focused on specific areas: two points at the right and left buccal shelf (RBF and LBF), two points at the right and left lingual flanges (RLF and LLF), two points at the right and left retromolar pad areas (RRP and LRP), and one point at the labial flange (LF). A color scale illustrated the adaptation between the denture base and the cast (Fig. 11). Following the standard metrology convention^40,41^, Blue hues (light to dark blue) represent negative deviation, indicating areas where the denture base is positioned inside the master cast surface (interference/pressure). Yellow to red hues represent positive deviation, indicating areas where the denture base is outside the cast surface (gap/space). Green represents minimal deviation within the defined tolerance zone. This data was also represented numerically, where positive values denoted gaps and negative values denoted pressure points. Additionally, the average deviation of the denture base from the cast was provided.

The data were analyzed using SPSS version 22 (SPSS Inc., Chicago, IL, USA), with the normality of distribution assessed by the Shapiro–Wilk test. For the peri-implant health indices (PI, GI, BI, PD), between-group comparisons (milled vs. printed) at each observation time were performed using the Mann–Whitney U test, while within-group changes across the three time points (T0, T6, T12) were analyzed with the Friedman test followed by post-hoc Wilcoxon signed-rank tests with Bonferroni adjustment. Denture base adaptation, measured at a single time point after fabrication, was compared between the two groups using an independent samples t-test. A p-value of less than 0.05 was considered statistically significant.

## Results

The baseline demographic and clinical characteristics of the participants are presented in Table 1. The two groups were comparable in all measured parameters at baseline (all *p* > 0.05). The results of clinical peri-implant soft tissue health for both groups at various observation times are presented in Table 2. No serious adverse events related to the interventions were reported during the trial. Both groups experienced a significant increase in Gingival Index (GI) and Plaque Index (PI) over time. While there was no significant difference between the measurements at T0 and T1, there were significant differences observed between T0 and T2, as well as between T1 and T2 for both groups. Throughout all observation periods, GI and PI did not significantly differ between the two groups. There was no significant difference in Bleeding Index (BI) between the different observation times for both groups, nor between the groups at any observation point. The Probing Depth (PD) significantly increased over time for the milled group only. At all observation times, there were no statistically significant differences in PD between the two groups. In the milled group, a significant difference was noted only between T0 and T2, with no significant differences observed between the other measurement times.

**Table 1:** Baseline characters for groups.

Characteristic	CAD/CAM Milled Group (*n* = 11)	3D-Printed Group (*n* = 11)	*p* value
Age (years)			
Mean ± SD	62.5 ± 8.2	64.1 ± 7.5	0.65
Range	45–75	48–80	
Gender, n (%)			
Male	7 (63.6%)	7 (63.6%)	1.00
Female	4 (36.4%)	4 (36.4%)	
Years of Edentulism			
Mean ± SD	10.2 ± 4.5	11.0 ± 5.1	0.72
Bone height in (mm)			
Mean ± SD	10.3–14 (11.7 ± 1.15)	11.5–15 (12.7 ± 2.03)	0.48

**Table 2:** Comparison of clinical outcomes between groups and observation times.

	T0 M (min-max)	T6 M (min-max)	T12 M (min-max)	Freidman *P* value
Plaque index (PI)				
Milled group	0.00 a (0.00–0.00)	0.00 a (0.00–1.00)	1.00 b (0.00–2.00)	< 0.001*
Printed group	0.00 a (0.00–0.00)	0.00 a (0.00–1.00)	1.00 b (0.00–2.00)	< 0.001*
Mann Whitney *P* value	1.00	0.551	0.133	
Gingival index (GI)				
Milled group	0.00 a (0.00–0.00)	0.00 a (0.00–0.00)	1.00 b (0.00–1.00)	< 0.001*
Printed group	0.00 a (0.00–0.00)	0.00 a (0.00–1.00)	1.00 b (0.00–1.00)	< 0.001*
Mann Whitney *P* value	1.00	0.151	0.714	
Probing depth (PD)				
Milled group	. 0.12a (0.00-0.25)	0.50a, b (0.25-0.75)	1.12b (1.00-1.25)	0.010*
Printed group	0.12a (0.00-0.75)	0.50a (0.00-0.75)	1.12b (0.50 − 1.5)	0.068
Mann Whitney *P* value	0.752	0.765	1.00	
Bleeding index (BI)				
Milled group	0.00 a (0.00–0.00)	0.00 a (0.00–0.00)	0.00 a (0.00–1.00)	0.051
Printed group	0.00 a (0.00–0.00)	0.00 a (0.00–1.00)	0.00 a (0.00–1.00)	0.216
Mann Whitney *P* value	1.00	0.151	1.00	

Data presented as mean ± standard deviation. Different superscript lowercase letters (a, b, c) within a row indicate statistically significant differences over time (T0, T6, T12) according to the Friedman test followed by post-hoc Wilcoxon signed-rank tests with Bonferroni correction (*p* < 0.05). Identical letters within a row indicate no significant difference.

The comparison of denture base adaptation between the two groups and various surface areas is presented in Table 3. A significant difference in adaptation was observed between the groups for all areas except for the right buccal shelf (RBF) and labial flange (LF). The 3D-printed dentures exhibited significantly greater positive deviation (indicating pressure or interference) at the right and left retromolar pads (RRP and LRP) compared to the CAD/CAM milled dentures, which showed values closer to the master cast surface in these areas. In contrast, the milled groups exhibited significantly higher pressure areas than the printed groups for both the right and left lingual flange (RLF and LLF). Additionally, for the left buccal shelf (LBF), the milled groups had significantly better adaptation compared to the printed groups. Overall, there was a significant difference in adaptation across the entire surface area, with the milled group showing superior adaptation compared to the printed group.

**Table 3:** Comparison of denture base adaptation between different groups and different surface areas.

	Printed group		Milled group		T-test *P* value
	X	SD	X	SD	
RBF	.0364a	0.0246	.0399a	0.0043	0.900
LBF	.2565b	0.0130	.1143b	0.0150	< 0.001*
RRP	.3903c	0.0038	.0169a	0.0081	< 0.001*
LRP	.1932d	0.0536	.0788a, c	0.0142	0.001*
RLF	− .3561e	0.0139	− .0652d	0.0223	< 0.001*
LLF	− .2607f	0.0600	.1579e	0.0237	< 0.001*
LF	.0493a	0.0373	.0832a, f	0.0149	0.240
Whole surface area	0.08111	0.00637	0.02382	0.00697	0.013*
2-way ANOVA *P* value	< 0.001*		< 0.001*		

X; mean; SD; standard deviation, *p is significant at 5%. Different letters in the same column showed a significant difference between each 2 areas (Bonferroni test, *p* < 0.05), while similar letters indicate no significant difference.

## Discussion

The absence of statistically significant differences in peri-implant soft tissue parameters between the study groups supports the acceptance of the first null hypothesis. In contrast, the detection of a significant difference in denture base adaptation by differing the construction technique between groups warrants the rejection of the second null hypothesis.

The comparison between CAD/CAM milled and 3D-printed implant-assisted overdentures is a rapidly evolving area in prosthodontics, with both techniques offering distinct advantages. CAD/CAM milling is consistently associated with superior fit, flexural strength, and fracture resistance, leading to better tissue adaptation and potentially improved long-term durability and patient comfort compared to both conventional and 3D-printed methods^7,23,42,43^. However, 3D printing is gaining ground due to its ability to produce clinically acceptable prostheses with good adaptation, especially when optimized laboratory protocols are followed, and it offers advantages in terms of rapid prototyping and cost-effectiveness^22,42,43^.

According to the result of this study, the observed increase in Gingival Index (GI) and Plaque Index (PI) over time in both groups aligns with previous research, which consistently reports that implant-supported overdentures can lead to greater plaque accumulation and gingival inflammation as time progresses, regardless of fabrication method^44–46^. This can be attributed to the challenges of maintaining optimal oral hygiene around implant-supported overdentures, where the design and fit of the prosthesis, as well as patient cleaning habits, play significant roles^44,46^. Studies comparing milled and 3D-printed restorations generally find no significant difference in plaque accumulation between the two fabrication techniques. This suggests that both materials are similarly biocompatible and do not inherently increase the risk of peri-implant inflammation. This conclusion aligns with the current study’s results, as throughout all observation periods, the Gingival Index (GI) and Plaque Index (PI) did not show significant differences between the two groups. The increase in GI and PI over time is therefore not surprising and can be attributed to the inherent difficulty of cleaning around complex prosthetic structures, the patient’s oral hygiene practices, and the natural tendency for biofilm to accumulate on dental prostheses, regardless of whether they are milled or 3D-printed^44–46^. Regular professional maintenance and patient education remain crucial to minimize these effects and maintain peri-implant health.

A distinct and noteworthy finding from our study was the statistically significant increase in Probing Depth (PD) observed over time in the milled overdenture group, while the printed group demonstrated stable PD measurements. This outcome may seem counterintuitive, particularly given the superior denture base adaptation associated with milled prostheses. Nevertheless, the increase in PD must be interpreted within the context of the current diagnostic criteria for peri-implant health. According to the 2017 World Workshop consensus^47^, peri-implant health cannot be assessed based solely on PD. An increase in probing depth that occurs in the absence of signs of inflammation (such as bleeding or suppuration) and without radiographic evidence of bone loss does not indicate peri-implantitis^47,48^. In our research, the increased PD in the milled group did not coincide with a significant rise in bleeding indices or other clinical markers of active inflammation. This pattern suggests that the observed change may represent a non-pathological, biomechanical adaptation rather than a progression toward disease. Potential explanations for this increase include the greater rigidity of milled PMMA, which may influence the stress-strain distribution in the peri-implant tissues, resulting in a physiological soft tissue remodeling response^49–51^. This underscores the vital importance of conducting a multi-parameter assessment, including PD, Bleeding on Probing (BOP), and bone levels; rather than relying on a single metric when evaluating the health of peri-implant tissues.

One of the most critical parameters in evaluating prosthetic success is the degree of adaptation between the denture base intaglio surface and the master cast, as this directly influences retention, stability, and patient comfort. The comparison between milled and 3D-printed two-implant overdentures reveals nuanced differences in adaptation and pressure distribution across various anatomical regions. Several studies have highlighted that 3D-printed overdentures can achieve superior adaptation in specific areas, such as the retromolar pads and posterior palatal seal likely due to the precision of additive manufacturing and the ability to fine-tune printing parameters like build orientation, layer thickness, and resin polymerization protocols^11,42,52^. However, in the present study, the milled group demonstrated superior adaptation at the retromolar pads. Ali A.E.A. et al.^53^ demonstrated that 3D-printed overdentures exhibited more even occlusal force distribution compared to conventional techniques, suggesting improved tissue contact and load transfer.

The 3D printing process, as employed in this study, utilizes unpolymerized resin that requires post-print polymerization, introducing the potential for shrinkage and deformation^54^. To mitigate these effects, our protocol was carefully controlled. We used a Digital Light Processing (DLP) printer (Rasedent D50) with a layer thickness of 50 μm, a 405 nm LED light source, and a build angle of 0° (platform parallel to the build plate) to optimize the accuracy of both the fitting and polished surfaces. All prints used the manufacturer-recommended support structures and underwent a standardized post-processing protocol of washing in isopropanol and post-curing in a UV chamber for 15 min at 60 °C. These specific parameters: light intensity (inherent to the LED source), layer thickness, and build orientation, directly influence the geometric accuracy and mechanical integrity of the final prosthesis^55–57^ and were held constant to ensure consistency within the printed group. Digital Light Processing (DLP)-based systems, which utilize UV light and micromirror technology, allow for build angle customization, significantly impacting the fit and geometric accuracy of the final product. Moreover, layer thickness plays a role in prosthetic performance: a 100 μm thickness may enhance fitting surface accuracy, while 50 μm provides better polished surface detail. Notably, DLP printing might offer superior adaptability in replicating complex anatomical features compared to conventional milling methods^58^.

Conversely, milled overdentures, fabricated from pre-polymerized PMMA blocks, often demonstrate better adaptation in regions such as the mid-palatal area. The results for the buccal shelf in this study were mixed, showing no significant difference on the right side and better adaptation for the printed group on the left. This may be attributed to the dimensional stability and homogeneity of milled materials, as well as the subtractive nature of the process, which avoids polymerization shrinkage^42,43^. The enhanced fit of CAD-CAM milled dentures, as demonstrated in this study, can likely be attributed to the elimination of volumetric distortions typically associated with denture base fabrication. This improvement arises from the use of a fully pre-polymerized resin puck, processed under elevated temperature and pressure, from which the denture base is precisely milled using a subtractive manufacturing technique^7,54,59,60^. El-Shaheed et al.^7^reported that CAD/CAM milled overdentures exhibited significantly better tissue surface adaptation and higher maximum bite force compared to conventionally processed bases, reinforcing their clinical reliability. Moreover, strain gauge analysis by Gomaa^61^ indicated that milled overdentures induced lower deformation under functional loads, suggesting enhanced structural integrity.

Despite these distinctions, both fabrication methods have demonstrated clinically acceptable outcomes^62–64^. Studies such as those by Nabil et al.^63^, Zandinejad et al.^62^, and Srinivasan M et al.^64^ found no significant differences in masticatory performance or patient satisfaction between 3D-printed and milled overdentures, although esthetic preferences and wear resistance varied slightly. Additionally, while 3D-printed resins may exhibit slightly lower surface hardness and wear resistance over time, ongoing advancements in material science, such as the incorporation of nano-ZrO₂ particles, are addressing these limitations and improving mechanical performance^62,63,65,66^.

Ultimately, the findings of this study provide a more nuanced evidence base to guide the choice of fabrication method. Where maximum prosthetic adaptation and precision fit are the paramount clinical priorities, such as in cases with limited inter-arch space or demanding biomechanical conditions, the CAD/CAM milling technique may be favored^25,67^. Conversely, in clinical settings where digital infrastructure supports additive manufacturing and where the observed pattern of stable peri-implant probing depths is a valued biological parameter, 3D-printing presents a clinically sound alternative^68^. The availability of the respective digital workflow infrastructure remains a fundamental practical consideration^69^.

This study is limited by its short follow-up duration and relatively small sample size. Consequently, further research is recommended to incorporate randomized clinical trials with larger cohorts and extended observation periods to strengthen the evidence base. Additionally, it is recommended that subsequent investigations assess further clinical parameters, such as changes in alveolar bone height and patient-reported satisfaction, for both types of prosthetic designs evaluated.

## Conclusion

Within the limitations of this study, CAD/CAM milled implant-assisted mandibular overdentures demonstrated superior denture base adaptation compared to their 3D-printed counterparts. This finding underscores the precision achievable with subtractive manufacturing for definitive prostheses. Regarding the peri-implant tissues, both groups maintained comparable and stable levels of plaque control and gingival health throughout the observation period. A notable observation was a gradual increase in probing depth over time, specific to the milled group; however, this was not accompanied by increased inflammation or other signs of disease, suggesting it may reflect a non-pathological tissue response to the prosthesis. Therefore, while milling offers an advantage in prosthetic fit accuracy, both fabrication techniques appear clinically viable for maintaining peri-implant health, provided they are integrated with appropriate hygiene protocols and monitoring.

## Data Availability

The datasets used in the current study are available from the corresponding author upon request.
